# Combined QTL and Selective Sweep Mappings with Coding SNP Annotation and *cis*-eQTL Analysis Revealed *PARK2* and *JAG2* as New Candidate Genes for Adiposity Regulation

**DOI:** 10.1534/g3.115.016865

**Published:** 2015-02-03

**Authors:** Pierre-François Roux, Simon Boitard, Yuna Blum, Brian Parks, Alexandra Montagner, Etienne Mouisel, Anis Djari, Diane Esquerré, Colette Désert, Morgane Boutin, Sophie Leroux, Frédéric Lecerf, Elisabeth Le Bihan-Duval, Christophe Klopp, Bertrand Servin, Frédérique Pitel, Michel Jean Duclos, Hervé Guillou, Aldons J. Lusis, Olivier Demeure, Sandrine Lagarrigue

**Affiliations:** * INRA, UMR1348 Pegase, Saint-Gilles, 35590, France; † Agrocampus Ouest, UMR1348 Pegase, Rennes, 35000, France; ‡ Université Européenne de Bretagne, France; § UMR 7205 OSEB (MNHN–CNRS–EPHE–UPMC), Paris, 75005, France; ** INRA/AgroParisTech, UMR1313 GABI, Jouy-en-Josas, 78352, France; †† UMR1331, INP, UPS, TOXALIM, INRA, Toulouse, France; ‡‡ INSERM, UMR 1048 I2MC, 31432 Toulouse, France; §§ Department of Medicine/Division of Cardiology, UCLA, Los Angeles, California; *** INRA, SIGENAE, Castanet-Tolosan, 31326, France; ††† UMR INRA, INPT ENSAT, INPT ENVT-GENPHYSE, Castanet-Tolosan, 31326, France; ‡‡‡ INRA, UR83 Recherches Avicoles, Nouzilly, 37380, France; §§§ INRA, Plateforme GENOTOUL, Castanet-Tolosan, 31326, France; **** Department of Human Genetics, UCLA, Los Angeles, California

**Keywords:** selective sweeps, QTL, *cis*-eQTLs, divergent lines, adiposity, *JAG2*, *PARK2*

## Abstract

Very few causal genes have been identified by quantitative trait loci (QTL) mapping because of the large size of QTL, and most of them were identified thanks to functional links already known with the targeted phenotype. Here, we propose to combine selection signature detection, coding SNP annotation, and *cis*-expression QTL analyses to identify potential causal genes underlying QTL identified in divergent line designs. As a model, we chose experimental chicken lines divergently selected for only one trait, the abdominal fat weight, in which several QTL were previously mapped. Using new haplotype-based statistics exploiting the very high SNP density generated through whole-genome resequencing, we found 129 significant selective sweeps. Most of the QTL colocalized with at least one sweep, which markedly narrowed candidate region size. Some of those sweeps contained only one gene, therefore making them strong positional causal candidates with no presupposed function. We then focused on two of these QTL/sweeps. The absence of nonsynonymous SNPs in their coding regions strongly suggests the existence of causal mutations acting in *cis* on their expression, confirmed by *cis*-eQTL identification using either allele-specific expression or genetic mapping analyses. Additional expression analyses of those two genes in the chicken and mice contrasted for adiposity reinforces their link with this phenotype. This study shows for the first time the interest of combining selective sweeps mapping, coding SNP annotation and *cis*-eQTL analyses for identifying causative genes for a complex trait, in the context of divergent lines selected for this specific trait. Moreover, it highlights two genes, *JAG2* and *PARK2*, as new potential negative and positive key regulators of adiposity in chicken and mice.

The ultimate goal of quantitative trait loci (QTL) mapping approaches is to gain a better understanding of biological mechanisms involved in these phenotypes to offer molecular tools for medical diagnosis or animal selection. Over the past three decades, several thousand QTL have been mapped for different traits in many species. In this context, whereas genome-wide association study is developing as the reference QTL mapping method for human populations (Visscher *et al.* 2012; Li *et al.* 2012), most of the QTL referenced today in experimental or livestock species were mapped by linkage analysis approaches through F_2_ genetic crosses. This is especially the case for species such as chicken, for which the individual production is inexpensive; in this case, for each phenotype of interest, divergent lines are usually created to maximize QTL heterozygosity after crossing parental lines. To date, 4714 QTL have been referenced in the Mouse Genome Database (Eppig *et al.* 2012; Bult *et al.* 2013) and 8006, 8935, and 3923 QTL for cattle, pigs, and chickens, respectively, in the animal QTLdb database (Hu *et al.* 2013). The major weakness of QTL mapped with linkage analysis is their large size, usually in the range of several megabases (Mbs). These intervals may contain up to hundreds of genes, which impedes the identification of causal underlying genes (Georges 2007). When dealing with these large genomic regions and the numerous positional candidate genes they contain, it is tempting to focus narrowly on functional candidates. Hence, many of the causal genes identified to date were already known as being functionally related to the complex trait, limiting the research scope (Grisart *et al.* 2004; Clop *et al.* 2006; Le Bihan-Duval *et al.* 2011).

We attempted to combine selection signatures in divergent lines for one complex trait, *cis*-eQTL analysis and single nucleotide polymorphisms (SNPs) annotation in coding regions by using DNA-seq, to improve identification of causal genes underlying QTL regions with no presupposed function. The model we used is based on two chicken lines divergently selected for abdominal fat weight (AF), for which six QTL responsible for AF were previously mapped with an average size of 12 Mbs, and containing tens to hundreds of genes.

With the development of high-density marker genotyping, several studies aiming at detecting selection signatures have been conducted during the past decade. These studies generally focus on natural populations (Lao *et al.* 2007; Barreiro *et al.* 2008) or on livestock populations subjected to artificial selection performed on several traits of agronomical interest (Hayes *et al.* 2009; Rubin *et al.* 2010; Kijas *et al.* 2012), or characterize the impact of domestication on genetics of livestock species (Qanbari *et al.* 2014). To date, in the animal selection field, only two studies have explored selection signatures within two lines divergently selected for a unique trait. Both were based on chicken and adopted classical F_ST_ approaches underpinned by 60,000 SNP markers (Johansson *et al.* 2010; Zhang *et al.* 2012). In this study, we also propose detecting molecular selection signatures in two chicken lines divergently selected for one trait (*i.e.*, the abdominal fat weight), but we use millions of SNPs. Several approaches exist to detect selective sweeps at the population level. They are based on allelic frequency patterns within populations (Kim and Stephan 2002; Kim and Nielsen 2004; Nielsen *et al.* 2005; Boitard *et al.* 2009), on the structure of haplotypes segregating in populations measured by extended haplotype homozygosity (EHH) or on related statistics (Sabeti *et al.* 2002; Voight *et al.* 2006), or on genetic differentiation between populations measured by single marker statistics such as F_ST_ (Lewontin and Krakauer 1973; Beaumont and Balding 2004; Riebler *et al.* 2008; Foll and Gaggiotti 2008; Gautier *et al.* 2009; Bonhomme *et al.* 2010). This last approach is particularly well-suited to our application, where whole SNP data from divergently selected lines are available. To take advantage of this high density of markers, we used the recently developed statistical test hapFLK (Fariello *et al.* 2013), which measures genetic differentiation between samples based on haplotype rather than single marker allele frequencies and, thus, naturally accounts for the correlation structure between SNPs. The authors showed that hapFLK increases the power to detect selection compared with classical F_ST_-based or EHH-based approaches. It is also well-adapted to the analysis of small effective size populations, like the chicken lines considered in the study. The level of genetic drift resulting from population neutral history is first evaluated using genome-wide data, and genomic regions are detected under selection only if they exhibit haplotype frequency patterns that are very unlikely to arise from the drift process. Previous studies have shown that this strategy allows efficient control of the false-positive rate (Fariello *et al.* 2013, 2014) even in the case of bottlenecked populations.

Another important advantage in using DNA-seq data is the availability of almost all the polymorphisms, as indels and SNPs, characterizing individuals of interest. Also, access to this type of data allows the exhaustive analysis of polymorphisms within both coding and regulatory regions of positional causal candidate genes underlying QTL. Among those positional candidates, the availability of DNA-seq data allow discrimination of two kinds of genes: genes with SNPs or indels impacting mature protein—which directly reinforce their causal status—from genes for which polymorphisms may act in *cis* on their expression, which requires investigation of their expression in tissues in which they are expressed to emphasize their causal status. For revealing *cis*-eQTL, different approaches can be used (Babak *et al.* 2010; Montgomery and Dermitzakis 2011; Lagarrigue *et al.* 2013). It could consist of analyzing segregation in families (linkage analysis) or association in populations (GWAS) between markers and the expression of the gene considered. However, it is also conceivable to analyze if the gene considered exhibits allele-specific expression (ASE). ASE can be quantified using technology allowing estimation of the transcript level depending on a SNP specific of each chromosomal copy. It is important to notice that such an approach needs to focus on tissues in which we expect, for the gene considered, a significant expression and a key role in the complex trait of interest.

In our experimental design, the analysis of colocalization of QTL and selective sweeps markedly shortened the list of candidate genes in each region, sometimes even down to one, making the latter a strong causative positional candidate. We therefore focused on two genes, each located in two distinct QTL colocalizing with different selective sweep patterns. The absence of missense and nonsense SNP on those genes strongly suggested that their expression should be regulated in *cis*. We thus confirmed *cis* regulation in chicken or mice models, contrasted for adiposity by analyzing ASE (chicken) or GWAS (mice). These observations therefore strengthen positional and also functional status of those genes. By combining these complementary approaches, summarized in Figure 1, we identified two strong positional and functional candidate genes underlying AF QTL, including one previously unknown to be involved in the genetic control of adiposity.

**Figure 1:**
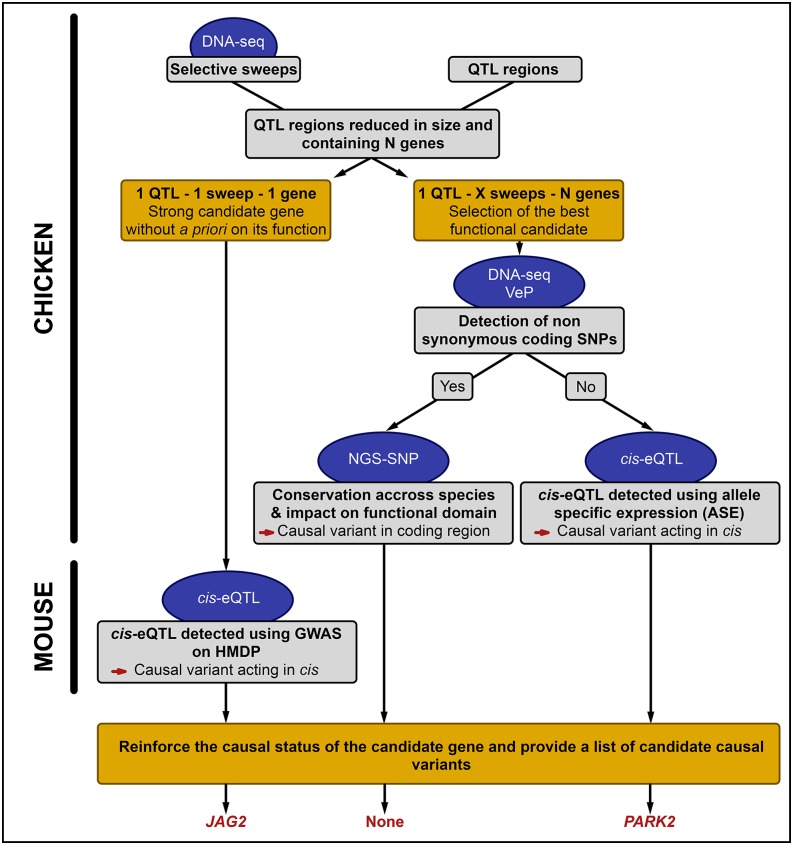
Overview of the strategy used in this study. QTL: quantitative trait locus; *cis*-eQTL: QTL for gene expression and acting in *cis*; ASE: allele-specific expression; VeP: Variant Effect Predictor tool from the Ensembl API; NGS-SNP: variant annotation tool developed by Grant *et al*. 2011; GWAS: genome-wide association study; HMDP: Hybrid Mouse Diversity Panel.

## Materials and Methods

### Chicken experimental design and ethics statement

The two experimental meat-type chicken lines were divergently selected for seven generations using the abdominal fat weight-to-animal weight ratio at 9 wk as an index of fattening, maintaining approximately similar live body weight (Leclercq *et al.* 1980). The two divergent lines were termed fat lines (FL) and lean lines (LL). After this selection, the two lines were maintained by just limiting inbreeding. Twenty animals from the 35^th^ generation were sequenced: seven LL, four FL, and nine F_1_ (FL × LL, unrelated animals) including the five F_1_ sires used for previous QTL study. Blood was collected from all the animals for DNA analyses. All procedures were conducted under License No. 37-123 from the Veterinary Services, Indre-et-Loire, France, and in accordance with guidelines for Care and Use of Animals in Agricultural Research and Teaching (French Agricultural Agency and Scientific Research Agency).

### Whole-genome DNA resequencing on chicken samples

DNA-seq paired-end libraries with a 250-bp insert were prepared using the TruSeq DNA Sample Preparation Kit (Illumina) according to the manufacturer’s instructions. The libraries were quantified using QPCR Library Quantification Kit (Agilent), checked on a High-Sensitivity DNA Chip (Agilent), and sequenced in pair end 2 × 101 bp on a HiSequation 2000 (Illumina) using a TruSeq v3 Kit. Sequencing produced, on average, 92% of raw reads correctly aligned against the reference genome per animal, *i.e.*, 20.4 Gb, corresponding to a sequencing depth of 19.7 X.

The read sets obtained by sequencing 20 animals were aligned against the *Gallus gallus* WASHUC2.1 genome reference obtained from Ensembl 58 using BWA v0.7.0 (Li and Durbin 2009). BAM files corresponding to samples sequenced on several lanes were then merged. All alignment BAM files were indexed and filtered. Only reads with a unique mapping hit and a minimal quality score of 30 were kept. PCR duplicates were filtered out using SAMtools rmdup. All these steps were performed using SAMtools v0.1.19 (Li *et al.* 2009). After all these filters, the mean sequencing depth decreased between 8× and 14×, depending on the chromosome. The reads cover approximately 86% of the genome, reaching 91% when unknown regions (stretches of Ns) are filtered out from the reference. The Genome Analysis Toolkit v2.4.9 software (GATK) (McKenna *et al.* 2010) was then used for realignment, recalibration, and variant calling. To minimize the number of mismatched bases across the reads, a local realignment was first performed around indel regions. A covariation analysis followed by a recalibration of base qualities was then conducted with GATK BaseQualityScoreRecalibrator. Finally, GATK UnifiedGenotyper was used for SNP and indel calling.

SNP validations were performed for the 16 SNPs in *JAG2* intronic regions. First, targeted sequences were amplified by PCR on 50 ng DNA using a Taq Uptitherm kit (Interchim) with the following primers: F 5′-ACCAGCAGATTCCAGTGCC-3′ – R 5′-GCCCCACTAAAACATGAGGG-3′. Amplicons (1817 bp) were then purified using a NucleoFast 96 PCR kit (Macherey Nagel) following the manufacturer’s protocol, and they were finally diluted at 50 ng/μL; 5 μL of each sample was then mixed with 5 μL of specific resequencing primers (Supporting Information, Table S1) and sent to GATC-Biotech (Konstanz, Germany) for Sanger resequencing. Sanger traces were analyzed using Mixed Sequence Reader (Chang *et al.* 2012).

### WGS-based selective sweep detection with hapFLK

We looked for genomic regions showing outstanding haplotypic differentiation between LL and FL using hapFLK statistics (Fariello *et al.* 2013), with a small modification to exploit the F_1_ data. The computation of hapFLK proceeds in three steps. First, a kinship matrix is estimated from pairwise genome-wide allele frequency differences between populations, which allows modeling of the extent of allele frequency differences that may result from genetic drift, and are thus compatible with a neutral evolution model. Second, genotypes from all sampled individuals are merged, and a pair of haplotype clusters is inferred for each individual at each SNP position using the algorithm developed by Scheet (Scheet and Stephens 2006) and used in the fastPHASE software. Haplotype clusters at one SNP position will be seen as ancestral haplotypes that summarize the genetic diversity in the neighborhood of the SNP. Third, sampled individuals are sorted again according to their population of origin, and cluster frequencies are computed at each SNP for all populations. The differentiation between populations at a given SNP position is then evaluated from the estimated cluster frequencies at this position using statistics related to a multi-allelic F_ST_, but it accounts for population structure through the previously estimated kinship matrix.

Here, we estimated the kinship matrix using SNP genotype data from the seven LL and the four FL samples; however, to infer haplotype clusters, we also included the nine F_1_ samples. F_1_ animals inherit their haplotypes from the LL and FL animals; therefore, including them allows an increase in sample size up to 20, and thus a more accurate inference of haplotype diversity.

Alleles that are rare in all populations are not informative for the detection of selection signatures; in the case of next-generation sequencing data, they may arise from sequencing errors. Consequently, we removed low-frequency SNPs (minor allele frequency below 10%) from hapFLK analyses. To speed-up the computations, we calculated the kinship matrix using only 1% of the high-frequency SNPs (estimating this matrix does not require a large number of SNPs) and computed hapFLK (with the modification mentioned above) using 30% of the high-frequency SNPs. When estimating haplotype clusters, we assumed six clusters, because this corresponds to the number of ancestral lines in the founder population, and estimated them using 10 expectation maximization iterations. We computed the *p*-value and the *q*-value associated with each hapFLK value following the procedure described by Fariello *et al.* (2013). In this procedure, the neutral distribution of hapFLK is first estimated from the hapFLK values observed genome-wide. Most loci in the genome are neutral. Besides, the few selected loci tend to produce outlier hapFLK values, which are automatically down-weighted by the statistical estimation procedure. The *p*-value at each SNP is then computed from the estimated neutral distribution so it does account for the effect of drift in the specific populations under study. Finally, SNPs with a *q*-value below 0.1 were considered under selection, which implies that 10% of these detected SNPs are expected to be false positives.

### Mice studies

To explore implications of highlighted candidate genes in adiposity, we focused on two mouse models, including strains highly variable for obesity-related phenotypes. The first, developed by Bennett *et al.* (2010) (Ghazalpour *et al.* 2012), named Hybrid Mouse Diversity Panel (HMDP), consists of a population of more than 96 inbred mouse strains selected for usage in systems genetics (QTL and eQTL) analyses related to complex traits (Farber *et al.* 2011; Calabrese *et al.* 2012; Ghazalpour *et al.* 2012). More details regarding the population can be found in Bennett *et al.* (2010) and Farber *et al.* (2011). The second includes B6.V-Lep^ob^/J mice (n = 8), null for the gene encoding leptin, and mice from the same genetic background C57BL6/J (n = 8). B6.V-Lep^ob^/J and HMDP mice were fed a standard chow diet and slaughtered at 14 and 16 wk old, respectively. The mice from the high-fat HMDP panel were fed high-fat diet during the last 8 wk.

### QTL and expression QTL analysis in HMDP

Population structure is a major confounder for genome-wide association analyses in the HMDP. This is due to the fact that many phenotypes correlate with the phylogeny of HMDP strains (*i.e.*, genetically similar strains have similar phenotypes), and any SNP that correlates with these strain relationships will be falsely associated with the phenotype. The Factored Spectrally Transformed Linear Mixed Models (FaST-LMM) algorithm has been shown to effectively reduce this confounding (Lippert *et al.* 2011). We applied this algorithm for mapping loci controlling either body adiposity mass (QTL) or transcript levels (eQTL) in mouse white adipose tissue (WAT). eQTLs are defined as *cis* if the peak SNP mapped within 1 Mb of the gene position (*p*-value threshold <10^−3^). The *cis* regulation indicates a potential functional genomic variation within or near a gene that significantly influences its expression.

### Differential expression analyses and tissue expression profiling

Total RNA from chicken (12 LL and 12 FL 9-wk-old individuals) and mouse liver and WAT were extracted using TRIzol reagent. RNA quality was assessed for each sample on an Agilent 2100 Bioanalyzer (Agilent Tech.). Reverse transcription was performed using the high-capacity cDNA archive kit (Applied Biosystems) according to the manufacturer’s protocol. Specific reverse and forward RT-qPCR primers are described in Table S2. Reaction mixtures were incubated in CFX96 Real-Time PCR Detector (Bio-Rad). The gene expression level was normalized relative to *GAPDH* expression level for chicken samples and relative to *HPRT* expression level for mouse samples using a ΔC_t_ approach. Reference and target genes had similar PCR efficiencies. The difference in expression for a gene was assessed using a Student *t*-test to compare 2^−ΔCt^ means between genotypes.

Procedures described for differential analysis were also used for tissue expression profiles in chicken, normalizing the target gene expression by using *18S* rRNA expression level. Pancreatic total RNA was prepared by the guanidinium thiocyanate extraction procedure developed by Chirgwin *et al.* (1979); for other tissues, total RNA was extracted with TRIzol reagent. Tissues with a C_t_ value above 30 were excluded from subsequent expression analysis, and related genes were considered as not expressed. Results are given as the gene expression fold change in each tissue relative to the tissue in which this gene was less expressed.

### Allele-specific expression characterization for *PARK2*

To investigate the allele-specific expression of *PARK2*, two marker SNPs, located on chromosome 3 at 46,581,638 bp and 46,581,695 bp on the reference genome WASHUC2.1, were tested on a Qiagen PyroMark Q24 sequencer in 8 F_1_ LL × FL chickens. Primers were designed using PyroMark Assay Design software (Table S3). gDNA and cDNA runs were analyzed by PyroMark Q24 1.0.10 software with default analysis parameters.

Only five F_1_ samples were further considered because the remaining three individuals were homozygous at both marker SNPs. For each sample, analyzed in duplicates, we standardized cDNA ratios according to the gDNA ratios for which a perfect balance is theoretically expected. We then divided frequency of the reference allele by the frequency of the alternative allele for each SNP and each individual to obtain the allelic ratio. The significance of the allelic imbalance was assessed using a Mann-Whitney nonparametric test to compare the observed allelic ratio with the expected one (equal to 1).

### Functional characterization of SNPs

SNP annotations were performed using Ensembl Variant Effect Predictor. With this application, the location of a SNP within a gene can be defined as outside of the gene, in the coding sequence, or in untranslated regions (UTR). For the SNP that is localized in the coding sequence, the functional impact can be determined (introduction of a stop codon, splicing modification, etc.). For further analyses on coding SNPs of special interest, the tool NGS-SNP was used, which enabled us to add meta-information about conservation between orthologous sequences (Grant *et al.* 2011).

## Results

### Study design

The first goal of this study was to identify selective sweeps using high-density SNP data generated by whole-genome resequencing in two meat-type chicken lines divergently selected for abdominal fat (AF) weight. The F_0_ foundation stock was a mix of six different meat lines used by breeders during the 1970s (Leclercq *et al.* 1980). The size of the mix population was limited, comprising 23 sires and 68 dams. The selection was then conducted on AF with similar body weight at 63 days of age over seven successive generations and produced the two divergent lines: the FL and the LL. We observed a plateau for the selection criteria in the FL from the fourth generation, and we observed a progressive decrease in adiposity during the seventh generation in the LL. Both lines were then maintained with 20 sires at each generation, without any decrease in the abdominal fat content divergence, which remained close to a factor of three at generation 35. For further selective sweep analyses, we collected blood from 11 F_0_ animals from FL and LL and from nine F_1_ (FL × LL) animals for genome resequencing.

### Genome-wide SNP identification in two divergent lines

Reads were aligned to the reference genome WASHUC2.1. In all, 92% of the total raw reads were correctly aligned and the raw coverage was, on average, 19.7 X ± 3.6 X. After the removal of PCR duplicates, and to limit the number of false positives due to sequencing errors or read multi-mapping, we retained only SNPs detected from reads that could be uniquely mapped with mapping quality scores higher than 30. After these filtering steps, 86% of the genome was covered, and the sequencing depth was 12.6 X ± 4.6 X. Subsequently, the SNP calling was performed using the GATK software, which includes modules for sequence realignment and recalibration reducing biases due to sequencing or sample preparation. A total of 11,847,150 SNPs were identified, of which 9,422,311 (79.5%) had genotype information for all the 20 animals (*i.e.*, call rate of 100%). Sanger resequencing was used to validate a set of 17 SNPs. The insertions and deletions (indels) were also detected: a total of 1,090,316 indels were identified, of which 810,754 (74.4%) had genotype information for all the animals.

Among the 9,422,311 SNPs observed in the whole population (*i.e.*, 20 birds), an average of 2,729,525 SNPs were identified within a single bird, corresponding to a density of 2.6 ± 0.5 SNP/kb. These results are in agreement with previous studies (International Chicken Genome Sequencing Consortium 2004; Wong *et al.* 2004). As expected, SNPs were mainly found in intergenic (48.7%) and intronic (41.8%) regions, rather than in regulatory regions (8.4%) and coding regions (1.2%) (File S1). In coding regions, SNPs associated with a synonymous annotation (59.45%), SNPs leading to amino acid changes (25.24%), SNPs leading to a gain or a loss of a start or stop codon (0.45%), and SNPs corresponding to a splicing site (14.83%) (Figure S1) were set apart.

### Identification of selective sweeps using hapFLK with 9.4 M SNPs in two divergent lines

The 9.4 million SNPs generated by whole-genome resequencing were used to detect genomic regions showing outstanding haplotypic differentiation between LL and FL. To do so, we used the hapFLK statistical test recently developed by Fariello *et al.* (2013) that is well-adapted to deal with millions of SNPs and to analyze populations with small effective size, like the chicken lines considered in our study. The genome scan performed with hapFLK revealed 129 significant selective sweeps (Table 1). These regions were rather evenly distributed across the genome, had an average size of 97.54 kb, and contained, on average, 838 SNPs and 2.11 genes each (Table 1). Given that sweep regions were not only shaped by the first 7 generations of selection but also by the next 28 generations of neutral evolution, this average sweep size of 100 kb is consistent with the approximate expectation of 1 / (*n* × *ρ* × *T*) = 95 kb, where n = 10 is the haploid sample size, *ρ* = 3 × 10^−8^ is the bp per generation recombination rate in chicken (Wong *et al.* 2004; Groenen *et al.* 2011), and *T* = 35 is the total number of generations. Focusing on the 52 sweeps containing only one gene, we performed a gene set enrichment analysis based on functional annotations. Among those 52 genes, this analysis revealed a significant enrichment for genes related to lipid metabolism (*P* < 10^−14^, χ^2^ test).

**Table 1:** Genome-wide description of selective sweeps identified using hapFLK

			**Selective Sweeps Size**				**SNPs/Sweep**			**Genes/Sweep**			
**Chr**	**Chr Size (Mb)**	**Selective Sweeps (*n*)**	**Mean (kb)**	**Min (kb)**	**Max (kb)**	**Coverage (%)**	**Mean (*n*)**	**Min (*n*)**	**Max (*n*)**	**Mean (*n*)**	**Min (*n*)**	**Max (*n*)**	**Total (*n*)**
**1**	200.99	36	127.28	1.74	789.46	2.22	1348.37	18	10,096	1.69	0	8	61
**2**	154.87	25	89.55	0.08	505.77	1.45	787.00	5	5238	1.64	0	13	41
**3**	113.66	17	93.20	0.22	535.04	1.23	943.65	9	5336	1.47	0	6	25
**4**	94.23	21	124.44	0.75	754.56	2.91	1324.00	16	7583	2.71	0	32	57
**5**	62.24	1	1.29	1.29	1.29	0.002	16.00	16	16	1.00	1	1	1
**6**	37.40	5	41.12	17.31	74.93	0.55	409.00	201	961	2.00	0	5	10
**7**	38.38	3	272.65	206.99	393.85	2.13	2724.70	2530	3019	2.67	2	4	8
**8**	30.67	1	103.19	103.19	103.19	0.34	570.00	570	570	2.00	2	2	2
**10**	22.56	6	11.04	0.10	46.48	0.29	142.00	7	555	2.17	1	6	13
**11**	21.93	5	303.97	10.10	1018.14	6.93	1623.60	86	4532	3.00	0	5	15
**12**	20.54	1	15.86	15.86	15.86	0.08	94.00	94	94	0.00	0	0	0
**13**	18.91	1	0.01	0.01	0.01	0.00	2.00	2	2	1.00	1	1	1
**14**	15.82	4	112.17	22.41	166.90	2.84	762.75	186	1620	5.00	4	6	20
**17**	11.18	1	107.06	107.06	107.06	0.96	650.00	650	650	1.00	1	1	1
**20**	13.99	1	151.00	151.00	151.00	1.08	1895.00	1895	1895	6.00	6	6	6
**24**	6.40	1	6.81	6.81	6.81	0.11	114.00	114	114	1.00	1	1	1
**Total**	1050.9	129	—	—	—	1.36	—	—	—	—	—	—	262
**Mean**	—	—	97.54	—	—	—	837.88	—	—	2.11	—	—	—

This approach, based on outstanding haplotypic differentiation between FL and LL, revealed the genomic regions being most impacted by the selection pressure applied on the common ancestral line. Because it is possible that some false-positive signals remain due to genetic drift among these 129 selective sweeps, we proposed to overlap those selective sweeps with AF QTL previously detected by linkage analysis to focus on the most compelling genomic regions.

### Overlapping QTL with selective sweep regions

**Figure 2:**
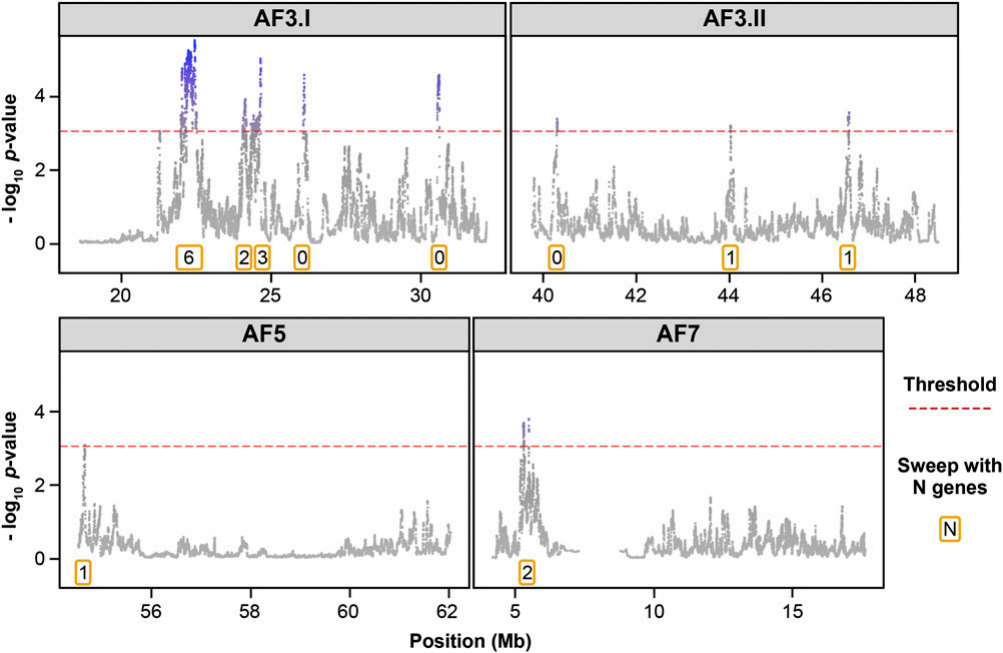
HapFLK statistics profile inside each AF QTL region. The associated name of each QTL region is given at the top of each graph. Statistics are written as the -log_10_ of the *p*-value of the HapFLK test; for each sweep, the number of genes it contained is indicated in an orange box. Some QTL (AF3.I and AF3.II) colocalized with several selective sweeps, whereas others (AF5, AF7, and BMWT1) colocalized with a single selective sweep. The number of genes included in a sweep ranges from 0 to 6.

In previous genetic studies of the LL and FL lines, we reported two QTL (*P* < 0.05) and four suggestive QTL (*P* < 0.1) for AF weight on chromosomes 1, 3, 5, and 7 using a F_0_-F_1_-F_2_ design generated by crossing the FL and LL lines (Lagarrigue *et al.* 2006). The design was composed of 5 F_1_ sire families with a total of 585 F_2_ offspring. The five F_1_ sires were included in the 20 animals we sequenced in this study at the whole-genome scale. Because it usually happens when using linkage analysis methods, QTL were resolved to a rather large genomic region, with a size ranging from 7.5 Mb to 18.1 Mb (Table 2). Four out of these six QTL colocalized with at least one selective sweep (Table 2). We also showed two-fold highly significant enrichment in selective sweeps within QTL compared with the genome level: 1.36% of the whole-genome against 2.48% of the total QTL intervals were covered by selective sweeps (*P* < 10^−16^, χ^2^ test). This clearly indicates that the relatively large number of sweeps detected genome-wide are not due to an excess of false positives, but rather to other factors related to trait architecture. As shown in Figure 2, sweep analysis revealed the genetic complexity underlying some QTL. The two QTL, AF3.I and AF3.II, on chromosome 3 colocalized with five and three selective sweeps, respectively. Conversely, the two QTL, AF5 and AF7, on chromosomes 5 and 7, respectively, had a simpler genetic profile and contained only one sweep with one and two genes, respectively (Figure 2 and Table 2). This approach combining selective sweep analysis and QTL mapping allowed a reduction in the size of the four QTL regions from, on average, 12 Mb to 100 kb, with some of these regions now containing one gene, therefore standing as strong causal positional candidate. Thus, this broad reduction of QTL size provided a great advance for the identification of causal genes. Subsequently, we set out to explore in greater depth two QTL to identify candidate causal underlying genes. With this aim, different data and approaches were combined.

**Table 2:** Characterization of selective sweeps colocalizing with QTL for abdominal fat

**QTL**							**Selective Sweep**				
**ID**	**Chr (*n*)**	**Start (Mb)**	**End (Mb)**	**Size (Mb)**	**Genes (*n*)**	***p****^a^*	**ID**	**Start (Mb)**	**End (Mb)**	**Size (Mb)**	**Genes***^b^*
							**AF3.I-a**	22.00	22.50	0.50	6
							**AF3.I-b**	24.03	24.16	0.13	2
**AF3.I**	**3**	18.61	32.15	13.54	171	$	**AF3.I-c**	24.30	24.64	0.34	3
							**AF3.I-d**	26.05	26.10	0.04	0
							**AF3.I-e**	30.52	30.59	0.07	2
							**AF3.II-a**	40.28	40.30	0.02	0
**AF3.II**	**3**	39.74	48.52	8.78	107	$	**AF3.II-b**	44.03	44.04	0.01	*MLLT4*
							**AF3.II-c**	46.57	46.60	0.03	*PARK2*
**AF5**	**5**	54.52	62.03	7.51	142	**	**AF5**	54.65	54.65	0.00	*JAG2*
**AF7**	**7**	4.21	17.63	13.42	229	$	**AF7**	5.31	5.52	0.21	2
**Mean**	—	—	—	*12.28*	*170*	*—*	*—*	—	—	*0.14*	*1.64*

a Chromosome-wide significance: **1%; *5%; $10% (suggestive QTL).

b Number of genes contained in each sweep or gene name for sweep containing only one gene.

### Focus on a QTL colocalizing with one sweep containing one gene revealed *JAG2* as a strong causal candidate for adiposity

**Figure 3:**
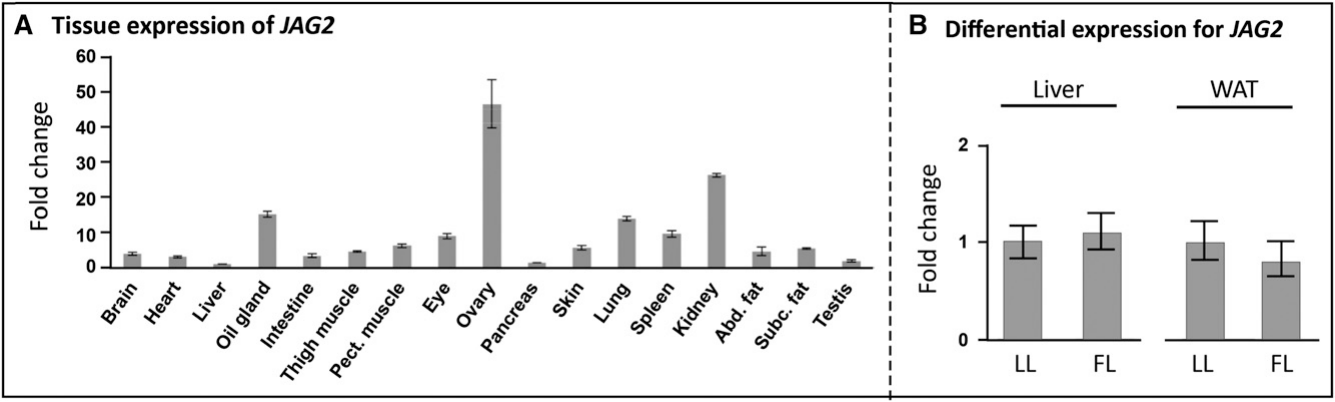
Expression characterizations of *JAG2* in chicken. (A) Expression pattern of *JAG2* in various tissues quantified using RT-qPCR. Results are given as expression fold change relative to the liver, which exhibited the lowest level of expression. (B) Comparison of *JAG2* mRNA level in the fat line (FL, n = 12) and the lean line (LL, n = 12) in the liver and the white adipose tissue (WAT). Results are expressed as the expression ratio relatively to the LL ± SEM.

The AF5 QTL was of great interest because it included a single sweep, with hapFLK statistics values clearly exceeding those observed in the rest of the QTL (Figure 2). This very short sweep, measuring 1.3 kb and termed AF5, contained only a portion of the gene *JAG2* (introns 17 to 19 and associated exons) (Figure S2). We did not find any indel or nonsynonymous SNPs suggesting the existence of causal polymorphism acting on the expression of this gene. Because liver and adipose tissue are major actors in lipid metabolism, we therefore focused on those tissues. We did not observe any significant differential expression between FL and LL in liver and adipose tissue (Figure 3B). We can hypothesize that *JAG2* expression could be regulated in *cis* in other tissues to influence obesity in chicken because *JAG2* is expressed in many tissues (Figure 3A), or that the age of birds we analyzed is irrelevant if there is any impact of developmental state on *JAG2* expression. Nevertheless, because *JAG2* is the only gene located in the sweep and colocalized with AF5 QTL, it is still a strong positional candidate gene for adiposity regulation.

**Figure 4:**
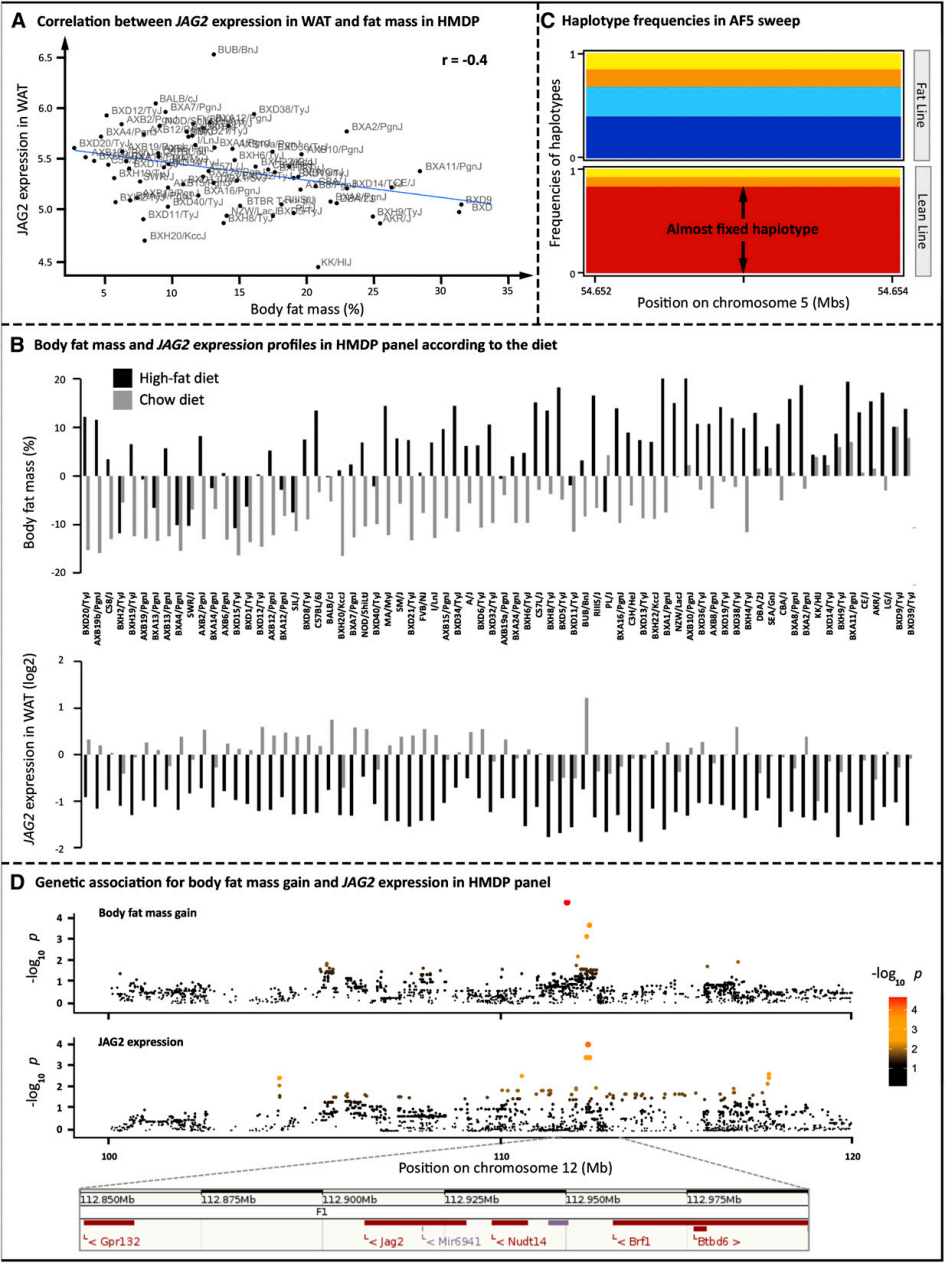
Expression of *JAG2* in mice HMDP. (A) Correlation between *JAG2* expression in white adipose tissue (WAT) and body fat mass (%) in HMDP of 86 mice fed a chow diet. (B) Body fat mass in % of body weight (top) and WAT expression (bottom) in HMDP panel of 86 mice fed chow diet (in grey) and high-fat/high-sucrose diet (in black). For each graph, values are centered on the mean of the whole values. (C) Haplotype cluster frequencies for both chicken lines for the *PARK2* selective sweep. The difference in color along the Y-axis gives the frequencies of each haplotype cluster. The difference in color along the X-axis has no meaning. The almost fixed haplotype is colored red in the lean line. (D) Genetic association (top) for body fat mass gain between the second and the fourth week of high-fat diet in the HMDP and (bottom) for *JAG2* expression in WAT. Gene positions as given in Ensembl 77 for GRCm38 mouse genome assembly .

To further question the implication of *JAG2* in adiposity, we studied its expression in liver and white adipose of two independent mice models contrasted for adiposity: B6.V-Lep^ob^/J mice (n = 8), which are KO for the gene encoding leptin and have severe obesity compared with mice from the same genetic background C57BL6/J (n = 8), and the Hybrid Mouse Diversity Panel (Bennett *et al.* 2010; Ghazalpour *et al.* 2012), which includes a population of 96 inbred mouse strains variable for obesity-related phenotypes, including total body fat mass. *JAG2* is lowly expressed in liver, contrarily to WAT in these two models. In WAT, no differential expression was observed between B6.V-Lep^ob^/J mice and C57BL6/J mice, whereas we identified a significant negative correlation between body fat mass and *JAG2* expression in WAT (Figure 4A; r = −0.4, *P* = 3 × 10^−8^, Pearson correlation test) in HMDP fed a chow diet. In addition, the difference of *JAG2* expression between chow and high-fat/high-sucrose diet was negatively correlated to the difference of fat mass between those diets (Figure 4B), highlighting a negative correlation between *JAG2* expression in WAT and adiposity induced by diet. HMDP mice fed a high-fat/high-sucrose diet are fatter than those maintained on a chow diet (top of Figure 4B), whereas *JAG2* expression in WAT is higher in chow diet compared with the high-fat/high-sucrose diet (bottom of Figure 4B). These results strengthen that *JAG2* is a negative regulator of adiposity. Therefore, because the *JAG2* haplotype was fixed under selection in the LL and not in the FL (Figure 4C), and because *JAG2* appears as a negative regulator of adiposity in mouse models, this selected haplotype should correspond to a “gain-of-function” mutation in *JAG2*. This result is also consistent with the direction of allele effects in the QTL mapping study reported by Lagarrigue *et al.* (2006), in which the microsatellite allele of the QTL associated with a decrease of adiposity came from the F_0_ lean line. To further explore the causal link between *JAG2* and adiposity, we performed a genetics association for *JAG2* expression and body fat mass in the HMDP design. As shown in Figure 4D, this analysis revealed a *cis*-eQTL for *JAG2* expression in adipose tissue that colocalized with a QTL for body fat mass gain (during the first weeks of high-fat diet). These results suggest that a mutation acting in *cis* on *JAG2* expression could be the causal mutation responsible for adiposity variation in this mice panel.

### Focus on a more complex QTL colocalizing with sweeps with two genes, *MLLT4* and *PARK2*

**Figure 5:**
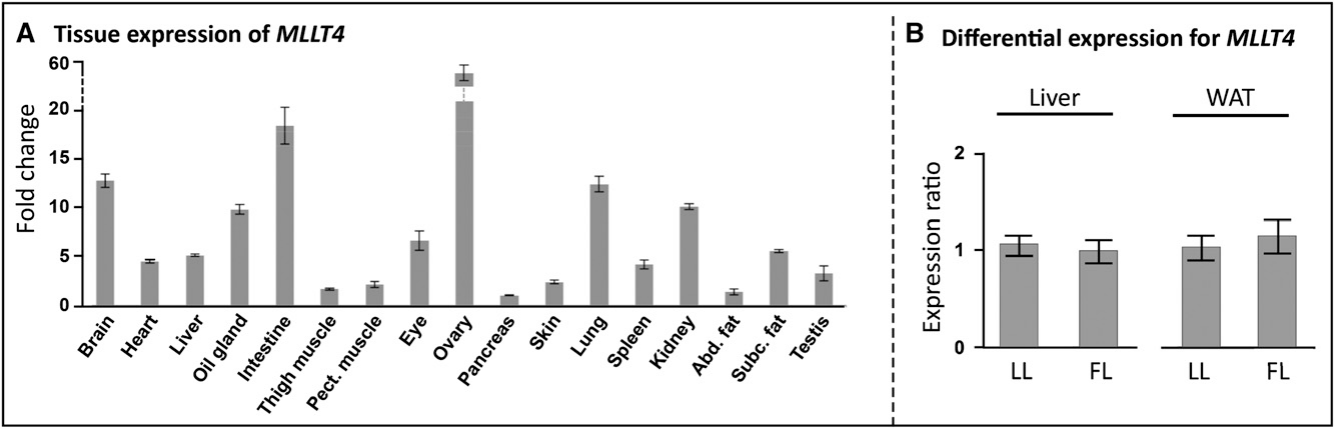
Expression of *MLLT4* in chicken. (A) Expression pattern of *MLLT4* in various tissues of chicken quantified using RT-qPCR. Results are given as expression fold change relative to the pancreas, which exhibited the lowest level of expression. (B) Comparison of *MLLT4* mRNA level in the fat line (FL, n = 12) and the lean line (LL, n = 12) in the liver and the white adipose tissue (WAT). Results are expressed as the expression ratio relative to the LL ± SEM.

**Figure 6:**
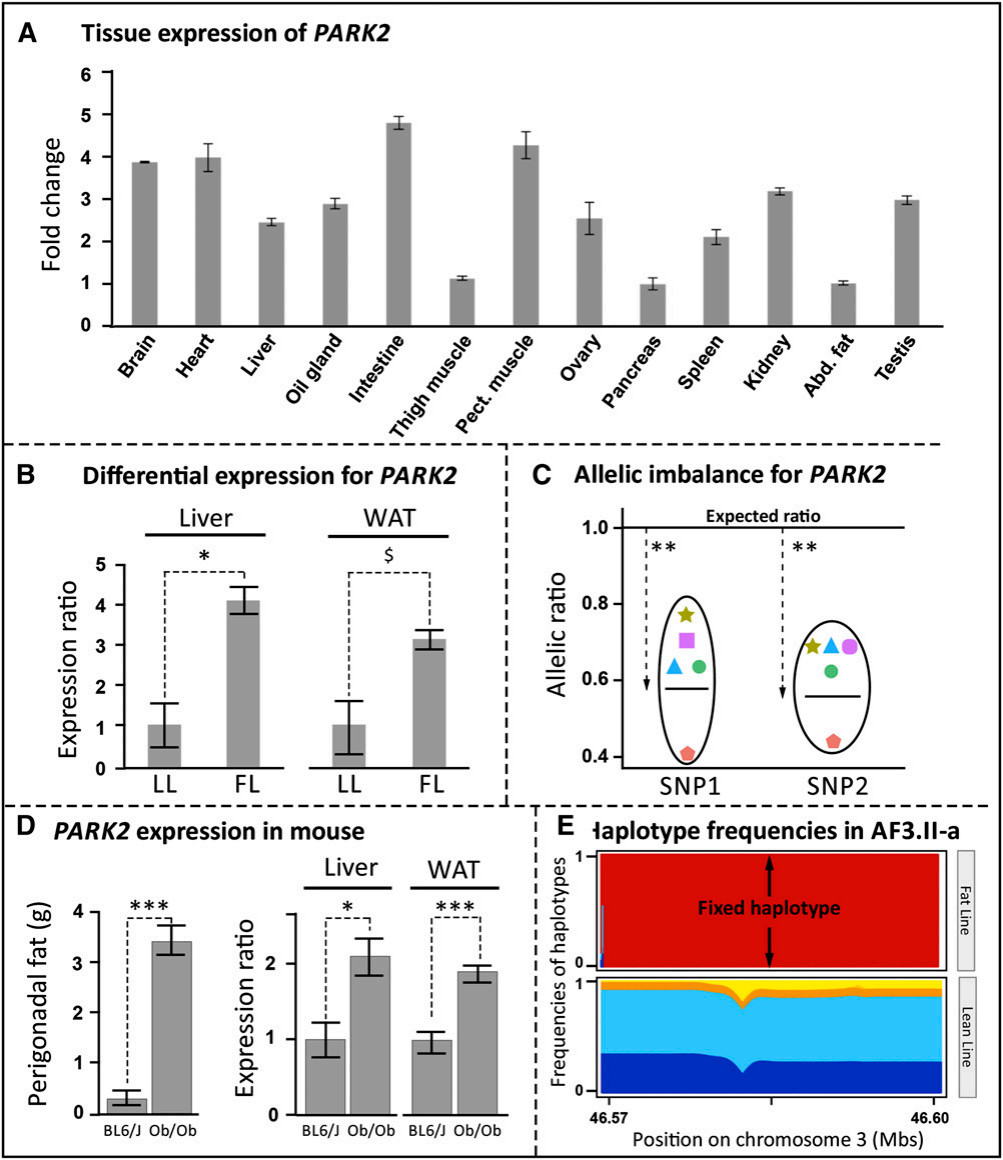
Expression characterizations of *PARK2*. (A) Expression pattern in various tissues of chickens quantified using RT-qPCR. Results are given as expression fold change relative to the tight muscle, which exhibited the lowest level of expression. (B) Comparison of *PARK2* mRNA level in the fat line (FL, n = 12) and the lean line (LL, n = 12) in the liver and the white adipose tissue (WAT). Results are expressed as the expression ratio relative to the LL ± SEM.*p < 5% and $*p <* 10% based on unpaired two-tailed Student *t*-test. (C) cDNA allelic ratio for two marker SNPs located on *PARK2* for F_1_ birds obtained by crossing chicken FL and LL. SNP_1_ is located on chromosome 3 at 46,581,638 bp and SNP_2_ is on chromosome 3 at 46,581,695 bp. A total of five birds heterozygous on gDNA at those positions were considered for pyro-sequencing-based cDNA imbalance analyses. Each color and shape is associated with one individual. Down arrows indicate the average allelic ratio for each SNP. The top line stands for the expected allelic ratio in the case of a perfect cDNA allelic balance. The line included in the circle stands for the average value of the imbalance for a given SNP for five individuals. Significance of the allelic imbalance was assessed using a Mann-Whitney unpaired two-tailed nonparametric test to compare the observed allelic ratio with the expected one equal to 1 (*i.e.*, in the case of a perfect balance). **p < 0.01. (D) Comparison of perigonadal fat mass (g) and *PARK2* mRNA level in the liver and the white adipose tissue (WAT) of B6.V-Lep^ob^/J mice (Ob, n = 8), KO for the gene encoding leptin, and their genetic background C57BL6/J (BL6, n = 8). For mRNA levels, results are expressed as the expression ratio relatively to the C57BL6/J ± SEM. *p < 0.05 and ***p < 0.001 based on unpaired two-tailed Student *t*-test. (E) Haplotype cluster frequencies for both chicken lines for the *PARK2* selective sweep. The difference in color along the Y-axis gives the frequencies of each haplotype cluster. The difference in color along the X-axis has no meaning. The fixed haplotype is red here in the FL.

Having established that our approach had sufficient resolution to target a unique sweep containing a unique gene, and to identify strong candidate genes underlying QTL with a simple genetic pattern, we then worked on deciphering the more complex QTL region AF3.II. As suggested by the hapFLK profile (Figure 2), this QTL region includes three selective sweeps, AF3.II-a, AF3.II-b, and AF3.II-c, one of them presumably containing a causative mutation. Those three sweeps contained, respectively, no gene, a fraction of *MLLT4* (from 1 kb upstream of the gene to intron 6), and a fraction of *PARK2* (the whole exon 3 and the flanking intronic regions) (File S2). We first focused on the sweep AF3.II-a containing no gene and established that there was no unannotated gene on this region that was expressed by visualizing, at the sweep position, all tissues RNA-seq reads available in Ensembl. Also, this sweep was located quite far from the closest annotated genes, *i.e.*, 80 kb upstream of *SLC35F3* and 100 kb downstream of *KCNK1*. Taken together, these results were strong enough to obviate a deeper exploration of the AF3.II-a sweep, with no clear evidence that this sweep was actually carrying a variant with an impact on AF. We then explored both sweeps AF3.II-b and AF3.II-c containing, respectively, a fraction of *MLLT4* and a fraction of *PARK2*. Focusing on polymorphisms in coding regions, we observed no indels or nonsynonymous SNPs, suggesting the existence of a causal polymorphism acting in *cis* on the expression of these genes. Regarding *JAG2*, we therefore focused on the expression of these two genes in hepatic and adipose tissues in both FL and LL lines. First, we verified that these genes are expressed in these tissues (Figure 5A and Figure 6A). We then analyzed differential expression of these two genes in those tissues between 12 FL and 12 LL. For *MLLT4*, this analysis revealed no differential expression (Figure 5B). For *PARK2*, which appeared as ubiquitously expressed with a high expression values in liver (Figure 6A) as already reported in humans (Cesari *et al.* 2003), we showed a suggested differential expression in WAT and a significant differential expression in liver between FL and LL (Figure 6B). This gene was significantly more expressed in liver in FL compared with LL (23.5 ± 0.3 C_t_ in FL *vs.* 24.9 ±0.5 C_t_ in LL; *P* < 0.01). We then generated F_1_ birds by crossing FL and LL to investigate the ASE using pyro-sequencing focusing on two marker SNPs for which assignment of the line-of-origin was possible. We found that *PARK2* was a *cis*-eQTL; it exhibited an ASE profile in liver at both marker SNPs (LL/FL allelic ratio of 0.6 ± 0.1 for both marker SNPs; *P* < 0.01; n = 5) (Figure 6C), suggesting that those markers are in linkage disequilibrium with a variant acting in *cis* on *PARK2* expression in this tissue. Based on gDNA allelic frequencies at those two markers observed in F_0_ individuals, we concluded that the underexpressed haplotype was characteristic of LL. In summary, with differential RT-qPCR analysis and a pyro-sequencing-based ASE approach, we obtained consistent results. First, we showed that *PARK2* was significantly less expressed in liver in LL. Second, we showed that an LL-specific haplotype was significantly less expressed in F_1_ liver. These results are consistent with the direction of allele effects in the QTL mapping study reported by Lagarrigue *et al.* (2006), in which the microsatellite allele of the AF3.II QTL associated with a decrease of adiposity came from the F_0_ lean line. Those results point to a *cis*-acting variant impacting the expression of *PARK2* in the liver of divergent chicken lines, emphasizing the potential causal status of this gene for adiposity.

To question the correlation between *PARK2* and adiposity in another animal model, we compared its expression profile between the two same mice models used for *JAG2* study. No significant correlation was observed between adiposity and *PARK2* expression in liver and WAT of the HMDP mice fed a chow diet. However, RT-qPCR analysis showed a two-fold increase in *PARK2* expression in liver and WAT of obese B6.V-Lep^ob^/J mice compared with wild-type mice (Figure 6D), revealing that its expression is positively correlated with body fat mass in mouse, as already observed in chicken. Therefore, because the *PARK2* haplotype was fixed under selection in the FL and not in the LL (Figure 6E), and because *PARK2* is a positive regulator of adiposity, this selected haplotype should correspond to a “gain-of-function” mutation in *PARK2*.

### Identification of causal variants in regulatory regions remains difficult

When there are nonsynonymous mutations in a positional candidate gene, it is often possible to target only a few candidate polymorphisms to deeply study *in silico* their impact on the protein and thereby select the best candidate for validation using, for example, site-directed mutagenesis. Nevertheless, in our study, in sweeps colocalizing with AF QTL, no gene had nonsynonymous mutations in coding regions. Our results, in agreement with previously reported results (Edwards *et al.* 2013), suggest that causal mutations underlying QTL may occur more often in regulatory regions than in coding regions. Yet, the identification of causal variants in regulatory regions remains difficult. In our case, subsequent analysis of DNA polymorphisms in related sweeps revealed no indel but 16 SNPs and 286 SNPs for noncoding regions of *PARK2* and *JAG2*, respectively. To go further, we selected SNPs with heterozygous genotypes consistent with the heterozygous genotypes of F_1_ sires at each QTL, which led to the observation of seven SNPs and 106 in each gene, respectively. We finally performed the analysis of *cis*-elements using Genomatix MatInspector tools with stringent criteria to filter in SNPs susceptible to impact potential transcription factor binding sites (PTFBS). This analysis revealed that 3 SNPs and 16 SNPs had an impact on 4 PTFBS and 22 PTFBS for *PARK2* and *JAG2*, respectively. This illustrates that, in regulatory regions, it remains difficult to identify causative mutation among all candidates.

## Discussion

Although selective sweep analysis revealed genomic regions affected by selection, QTL mapping approaches allow detection of genomic regions impacting a specific complex trait. Thus, in the context of single trait divergently selected lines, some selective sweeps should overlap with QTL regions for this trait. Although the divergent selection is performed on one specific trait, it could also occur on other traits that are in balance with the trait on which the selection is applied (*e.g.*, breast muscle weight in our case), which explains why some sweeps—and not all—should overlap with QTL mapped for the first phenotype. This study is, to our knowledge, the first to investigate the genetics of complex traits using a combined QTL mapping approach and high-throughput-based selective sweep mapping in divergently selected lines. Whereas previous selective sweep studies in chicken focused on the characterization of the genome-wide effects of divergent selection on a specific phenotype (Johansson *et al.* 2010; Zhang *et al.* 2012), here we integrate QTL mapping and whole-genome-based selective sweep detection for the same phenotype, and also SNPs and expression analyses. The sweep detection was performed using the hapFLK method, which is adapted to analyze whole-genome resequencing data by processing haplotypes instead of single markers, able to efficiently distinguish drift from selection, and efficient for population-related data as well as for divergent lines (Fariello *et al.* 2013). The genome scan based on 9,422,311 SNPs detected in both lines revealed 129 selective sweeps with a size of 97.54 kb, on average. Although the studies of Johansson *et al.* (2010) and Zhang *et al.* (2012) cannot be strictly compared with ours because of differences in the number of selection generations (50 and 11 *vs.* 7), marker density (60 k *vs.* 9.4 M SNPs), and methods used to detect selective sweeps (line-specific alleles and EHH *vs.* hapFLK), the number of sweeps was quite similar (163, 108, and 129, respectively).

Overlying results from the selective sweep scan and QTL previously described in our experimental design revealed that four out of six QTL were colocalizing with sweeps. This approach markedly narrowed candidate regions in those QTL and allowed us to highlight three candidate genes underlying two QTL, AF5 and AF3.II.

Focusing on the QTL AF5, the only gene present in the sweep was *JAG2* that encodes the Notch ligand Jagged-2 involved in the ligand-induced cleavage and nuclear translocation of the Notch1 and Notch2 receptors. The Notch signaling pathway is a central cellular signaling pathway involved in many essential cellular functions, and it is known to impact embryogenesis, central nervous development, cardiovascular development, and also angiogenesis (Andersson *et al.* 2011; Guruharsha *et al.* 2012). As shown by the Jackson Laboratory, *JAG2* appears essential in development processes, as the homozygous Jag2^deltaDSL^ mutant mice from the B6.129S1-Jag2^tm1Grid^/J strain exhibit syndactyly and craniofacial defects causing perinatal lethality in a Notch-mediated manner (Jiang *et al.* 1998). Our study based on selective sweep detection strongly suggests a role for *JAG2* in adiposity, which has never been highlighted in previous studies because this gene was the unique positional gene candidate in the QTL/sweep region. After characterizing the SNPs on the corresponding selective sweep AF5, we did not find any nonsynonymous mutations within coding regions of the gene among the 20 resequenced birds, strongly suggesting the presence of a causal mutation underlying the QTL AF5 in regulatory regions and acting in *cis* in one of the tissues where the gene is expressed. Unexpectedly, the differential expression between the two prepubertal chicken FL and LL was not observed in the two tissues sampled, suggesting that the role of *JAG2* related to adiposity occurs in another tissue or at another development stage. In contrast to chicken, our results with the adult mouse HMDP panel revealed a *cis*-eQTL for adipose *JAG2* expression colocalizing with a body fat gain QTL. In addition, this expression of *JAG2* in WAT was negatively correlated to adiposity trait in two models, one in which adiposity variation is due to the genetic background (r = −0.4) and another in which adiposity variation is due to the diet (high fat *vs.* chow). These results observed in chicken and mouse HMDP and based on genetic and expression approaches reveal that *JAG2* is a highly confident positional and functional candidate for regulating adiposity as a negative regulator of this trait.

Finally, for the QTL AF3.II, we revealed *PARK2* as an outstanding candidate gene. As stated in the literature, this gene belongs to the E3 ubiquitin-protein ligase family that promotes polyubiquitination with subsequent proteosomal degradation and/or mono-ubiquitination and multi-ubiquitination with post-translational regulation of protein function and stability (Uhrig *et al.* 2008; O’Neill 2009; Yin *et al.* 2010). Whereas *PARK2* was associated by a genetic approach with an autosomal recessive juvenile form of Parkinson disease (Kitada *et al.* 1998), its central role in this pathology is not obvious in mice (Goldberg *et al.* 2003; Perez and Palmiter 2005), and its biological role remains poorly understood. Here, we showed by different approaches in different animal models that *PARK2* could be responsible for adiposity variation. Interestingly, other E3 ubiquitin ligase enzymes have been identified as involved in the modulation of lipid biology (Molero *et al.* 2004; Zelcer *et al.* 2009). As highlighted in this study, because this gene is differentially expressed in liver, suggesting a functional role in this tissue implied in lipid metabolism, it is both a positional and a functional candidate. More recently, one study conducted by Sack’s laboratory (Perez and Palmiter 2005) showed that *PARK2*^−/−^ mice are extremely resistant to weight gain induced by a high-fat and high-cholesterol diet (HFD). Otherwise, in response to HFD feeding, the *PARK2*^+/+^ mice showed a robust increase in *PARK2* mRNA level, in parallel with elevated lipid transport protein levels, increased hepatic insulin resistance, and steatohepatitis. In addition to this unique study reporting a link between *PARK2* and adiposity, our study shows that *PARK2* is: a strong positional candidate for adiposity in chicken, because it alone was found in a sweep colocalizing with an AF QTL; a functional candidate, because it is differentially expressed in liver and WAT between the FL and the LL chicken lines as well as between the obese B6.V-Lep^ob^/J mice and wild-type mice from the same genetic C57BL6/J background; and a probable positive regulator of adiposity because it is systematically overexpressed in the fat animals. In addition, we clearly showed by the ASE approach in F_1_ FL × LL chickens that the hepatic overexpression in fat line is due to a polymorphism acting in *cis* on its expression. Taken together, these results for mouse and chicken strengthen the hypothesis that *PARK2* is a positive regulator of fat metabolism.

This study shows for the first time the interest to combine *cis*-eQTL and selective sweep mappings in divergent lines selected for a specific trait for identifying causative genes. As shown in Figure 1, which summarizes the strategy used in our study, selective sweep detection using DNA resequencing enabled us to markedly reduce the size of some QTL for adiposity (from 12 Mb to 100 kb, on average). Some of those QTL included only one sweep with one gene, standing as strong causal candidates with no presupposed function. Also, DNA resequencing provides access to nearly all polymorphisms within these genes, enabling inference of the type of action of causal polymorphisms within the candidate genes, *i.e.*, affecting the protein sequence if nonsynonymous SNPs/indels are identified in coding regions *vs.* acting in *cis* on gene expression and thus located in regulatory regions when no nonsynonymous SNP was found in coding regions. In this latter case, *cis*-eQTL analysis can help to reinforce the causative status for adiposity of those genes, as shown for *JAG2* in mice and *PARK2* in chicken. Analyses of expression variations in the different fat and lean models clearly show that the two causative candidates, *JAG2* and *PARK2*, act on adiposity as negative and positive regulators, respectively. However, it remains difficult to identify the causal variant among all potential *cis*-acting SNPs of the two investigated candidate genes. In conclusion, in a research field where the identification of causal genes remains exceptional, our strategy led to the identification of two highly confident causal candidates, one of which was never related to the trait of interest (*JAG2*) and another one (*PARK2*) that was linked to this trait in a single study.

## 

## Supplementary Material

Supporting Information
