# Supplementary material for: Combined QTL and Selective Sweep Mappings with Coding SNP Annotation and cis-eQTL Analysis Revealed PARK2 and JAG2 as New Candidate Genes for Adiposity Regulation
Source: G3 (Bethesda). 2015 Feb 3;5(4):517–29. doi: 10.1534/g3.115.016865 (PMC4390568; doi:10.1534/g3.115.016865)
Supplement: Supporting Information [file supp_5_4_517__index.html]

Combined QTL and Selective Sweep Mappings with Coding SNP Annotation and cis-eQTL Analysis Revealed PARK2 and JAG2 as New Candidate Genes for Adiposity Regulation — Supporting Information 

# Combined QTL and Selective Sweep Mappings with Coding SNP Annotation and *cis*-eQTL Analysis Revealed *PARK2* and *JAG2* as New Candidate Genes for Adiposity Regulation

## Supporting Information for Roux *et al.*, 2015

**Files in this Data Supplement:**

- Supporting Information - Figures S1-S2 and Tables S1-S3 (PDF, 220 KB)
- Figure S1 - Annotation of the 9.4 million SNPs identified in the two lines using whole-genome re-sequencing. (PDF, 96 KB)
- Figure S2 - Allelic and haplotypic cluster frequencies in each sweep underlying QTLs. (PDF, 324 KB)
- Table S1 - Description of primers used for SNP validations by Sanger re-sequencing. (PDF, 65 KB)
- Table S2 - Description of primers used for RT-qPCR. (PDF, 65 KB)
- Table S3 - Description of primers used for pyro-sequencing-based allelic imbalance analyses. (PDF, 64 KB)
