# Supplementary material for: Combined QTL and Selective Sweep Mappings with Coding SNP Annotation and cis-eQTL Analysis Revealed PARK2 and JAG2 as New Candidate Genes for Adiposity Regulation
Source: G3 (Bethesda). 2015 Feb 3;5(4):517–29. doi: 10.1534/g3.115.016865 (PMC4390568; doi:10.1534/g3.115.016865)
Supplement: Supporting Information [file supp_g3.115.016865_016865SI.pdf]

G3 · Genes | Genomes | Genetics

Combined QTL and selective sweep mappings with coding SNP annotation and *cis*-eQTL analysis revealed *PARK2* and *JAG2* as new candidate genes for adiposity regulation

Pierre-François Roux[*,§,†], Simon Boitard[‡,], Yuna Blum[§§], Brian Parks[§§], Alexandra Montagner[††], Etienne Mouisel[‡‡], Anis Djari[***], Diane Esquerré[§§§], Colette Désert[*,§,†], Morgane Boutin[*,§,†], Sophie Leroux[†††], Frédéric Lecerf[*,§,†], Elisabeth Le Bihan-Duval[‡‡‡], Christophe Klopp[***], Bertrand Servin[†††], Frédérique Pitel[†††], Michel Jean Duclos[‡‡‡], Hervé Guillou[††], Aldons J. Lusis[§§,****], Olivier Demeure[*,§,†] and Sandrine Lagarrigue[*,§,†,1]**

[*] INRA, UMR1348 Pegase, Saint-Gilles, 35590, France
[§] Agrocampus Ouest, UMR1348 Pegase, Rennes, 35000, France
[†] Université Européenne de Bretagne, France
[‡] UMR 7205 OSEB (MNHN – CNRS – EPHE – UPMC), Paris, 75005, France
[**] INRA/AgroParisTech, UMR1313 GABI, Jouy-en-Josas, 78352, France
[§§] Department of Medecine/Division of Cardiology, UCLA, Los Angeles, USA
[††] UMR1331, INP, UPS, TOXALIM, INRA, Toulouse, France
[‡‡] INSERM, UMR 1048 I2MC, 31432 Toulouse, France
[***] INRA, SIGENAE, Castanet-Tolosan, 31326, France
[§§§] INRA, Plateforme GENOTOUL, Castanet-Tolosan, 31326, France
[†††] UMR INRA, INPT ENSAT, INPT ENVT-GENPHYSE, Castanet-Tolosan, 31326, France
[‡‡‡] INRA, UR83 Recherches Avicoles, Nouzilly, 37380, France
[****] Department of Human Genetics, UCLA, Los Angeles, USA

Corresponding author
Pr. Sandrine Lagarrigue
65, rue de Saint Brieuc, Bâtiment 18
35042 Rennes Cedex, FRANCE
Phone: + 33 2 23 48 59 59
Email: sandrine.lagarrigue@agrocampus-ouest.fr

DOI : 10.1534/g3.115.016865

All types of SNP	SNP N	SNP %	Genes N
Intergenic	4513004	48.68	-
Regulatory regions	776080	8.37	17352
Intronic	3873604	41.78	14087
Coding - Synonymous	65276	0.70	11905
Coding - Non Synonymous	43410	0.47	10676

Coding SNP	SNP N	SNP %	Genes N
Synonymous	65276	59.45	11905
Missense	27711	25.24	8214
Initiator or stop codon	495	0.45	461
Splicing site	16286	14.83	7155
Mature mi-RNA	34	0.03	30

Figure S1 Annotation of the 9.4 million SNPs identified in the two lines using whole-genome re-sequencing.

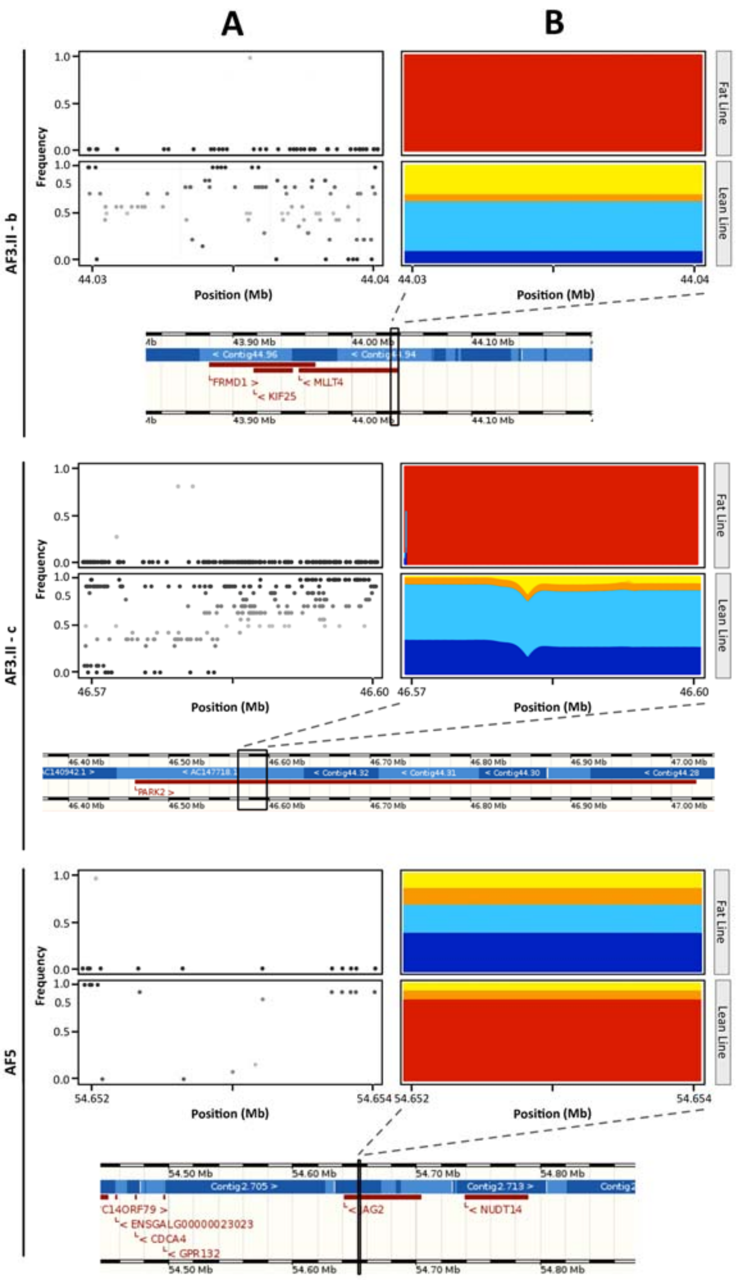

Figure S2 Allelic and haplotypic cluster frequencies in each sweep underlying QTLs.

(A) Allelic and (B) haplotypic cluster frequencies for both chicken lines in each selective sweep underlying QTL for abdominal fat weight (AF3.I, AF3.II, AF5). In A, the reference allele used for the SNP frequency calculation is arbitrary. In B, the difference in color along the Y-axis gives the frequencies of each haplotype cluster. The difference in color along the X-axis has no meaning. Almost fixed clusters are colored red. Under each graph, a genomic map gives the position of the sweep (in a black box) along the genome, and highlights genes inside or around the sweep (red).

Table S1 Description of primers used for SNP validations by Sanger re-sequencing.

ID	Sequence	Targeted strand	Start Position[1]	Length	Tm (°C)
**JAG2-Fwd1**	ACCAGCAGATTCCAGTGCCAC	+	1	20	64
**JAG2-Fwd2**	TCAGTATCTCATGTCAAGTGAC	+	411	22	58
**JAG2-Fwd3**	AGTACAAGAAATGCATGGTTCC	+	844	22	59
**JAG2-Fwd4**	ACAGTTCTTCTGGTTGTTAAGG	+	1271	22	59
**JAG2-Fwd5**	TGCTTCTCAGTCTTTCTTTCAC	+	1735	22	59
**JAG2-Rev1**	GCCCCACTAAAACATGAGGG	-	1	20	59
**JAG2-Rev2**	AAGCTAACTCCTTAACAACCAG	-	517	22	58
**JAG2-Rev3**	TGGCTTACTGTAAAGCATCAACTG	-	930	24	62
**JAG2-Rev4**	AATGTCTGGTCATAAGTAGCAG	-	1333	22	58
**JAG2-Rev5**	CAATCAAGCAATTGATCGTG	-	1759	20	56

[1] Relatively to the 3’ end of the targeted PCR amplicon.

Tables S2 Description of primers used for RT-qPCR.

ID	Species	Sequence (5' -> 3')	Length	Tm (°C)
**GG-JAG2-F**	*Gallus gallus*	AAATGCAATCACCAAGCGGC	20	65
**GG-JAG2-R**	*Gallus gallus*	ACCGCACAAAGAATTGGAACC	21	65
**GG-PARK2-F**	*Gallus gallus*	AGCACACCCAACAACTGACA	20	60
**GG-PARK2-R**	*Gallus gallus*	GAGTCTGGACAGCCAGCTAC	20	58
**GG-MLLT4-F**	*Gallus gallus*	ACTGCCACAACTCAGGATGT	20	58
**GG-MLLT4-R**	*Gallus gallus*	TTCAGGGCCATTACTCTGAGC	21	60
**GG-GAPDH-F**	*Gallus gallus*	GCTAAGGCTGTGGGGAAAGT	20	61
**GG-GAPDH-R**	*Gallus gallus*	TCAGCAGCAGCCTTCACTAC	20	60
**MM-PARK2-F**	*Mus musculus*	ATGAATCACAGCCTGCATTGTG	22	62
**MM-PARK2-R**	*Mus musculus*	TACCACCACACACACAGACTTC	22	60
**MM-HPRT-F**	*Mus musculus*	TGGCCATCTGCCTAGTAAAGC	21	62
**MM-HPRT-R**	*Mus musculus*	GGACGCAGCAACTGACATTTC	21	63

Table S3 Description of primers used for pyro-sequencing-based allelic imbalance analyses.

Chromosome	SNP position	Forward primer	Reverse primer	Sequencing primer
**3**	46581638	[BTN]--GTGCAGCTAGTTACAACAG	CCTTGCCAAAGTGAGTGTT	TTACATTCATTGCATCG
**3**	46581695	[BTN]--GTGCAGCTAGTTACAACAG	CCTTGCCAAAGTGAGTGTT	ATTCTTGCAAAAAACATAAA

[BTN]-- Biotinylated on 5' end.
