# Supplementary material for: Combined QTL and Selective Sweep Mappings with Coding SNP Annotation and cis-eQTL Analysis Revealed PARK2 and JAG2 as New Candidate Genes for Adiposity Regulation
Source: G3 (Bethesda). 2015 Feb 3;5(4):517–29. doi: 10.1534/g3.115.016865 (PMC4390568; doi:10.1534/g3.115.016865)
Supplement: Supporting Information [file supp_g3.115.016865_FigureS1.pdf]

All types of SNP	SNP N	SNP %	Genes N
Intergenic	4513004	48.68	-
Regulatory regions	776080	8.37	17352
Intronic	3873604	41.78	14087
Coding - Synonymous	65276	0.70	11905
Coding - Non Synonymous	43410	0.47	10676

Coding SNP	SNP N	SNP %	Genes N
Synonymous	65276	59.45	11905
Missense	27711	25.24	8214
Initiator or stop codon	495	0.45	461
Splicing site	16286	14.83	7155
Mature mi-RNA	34	0.03	30

Figure S1 Annotation of the 9.4 million SNPs identified in the two lines using whole-genome re-sequencing.
