# Supplementary material for: Combined QTL and Selective Sweep Mappings with Coding SNP Annotation and cis-eQTL Analysis Revealed PARK2 and JAG2 as New Candidate Genes for Adiposity Regulation
Source: G3 (Bethesda). 2015 Feb 3;5(4):517–29. doi: 10.1534/g3.115.016865 (PMC4390568; doi:10.1534/g3.115.016865)
Supplement: Supporting Information [file supp_g3.115.016865_FigureS2.pdf]

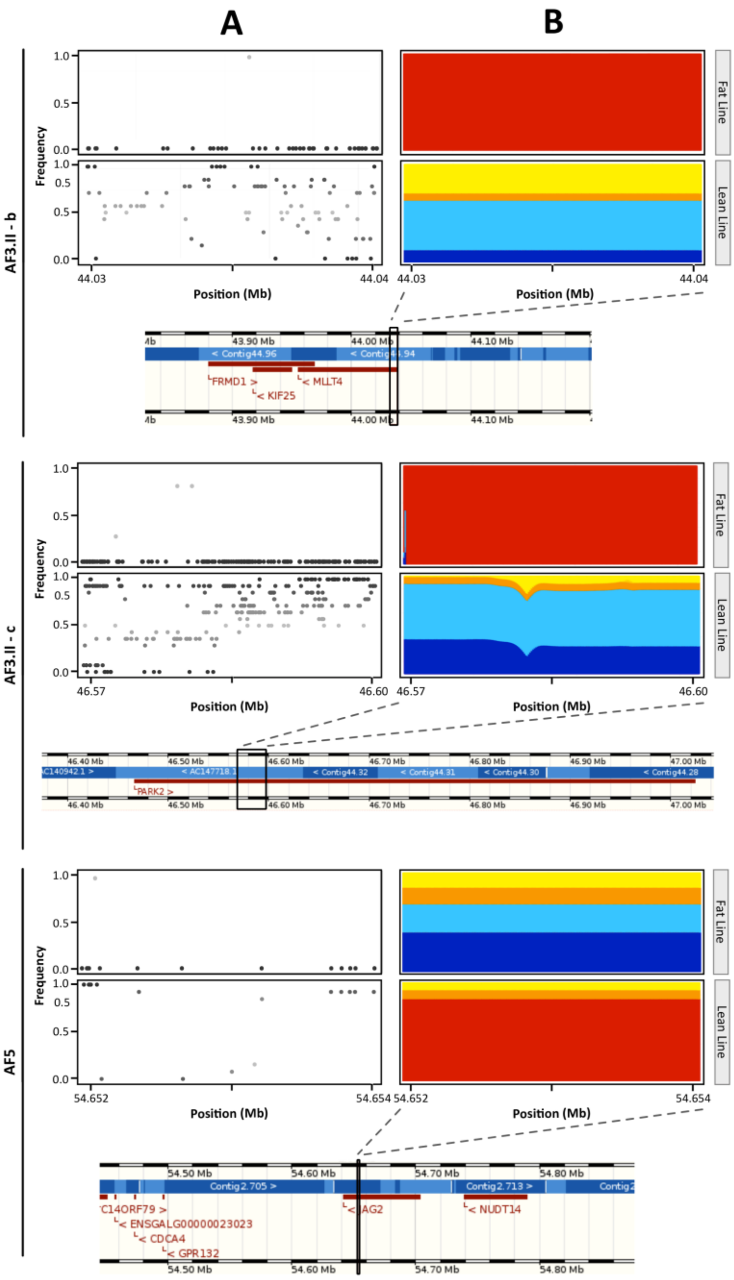

Figure S2 Allelic and haplotypic cluster frequencies in each sweep underlying QTLs.

(A) Allelic and (B) haplotypic cluster frequencies for both chicken lines in each selective sweep underlying QTL for abdominal fat weight (AF3.I, AF3.II, AF5). In A, the reference allele used for the SNP frequency calculation is arbitrary. In B, the difference in color along the Y-axis gives the frequencies of each haplotype cluster. The difference in color along the X-axis has no meaning. Almost fixed clusters are colored red. Under each graph, a genomic map gives the position of the sweep (in a black box) along the genome, and highlights genes inside or around the sweep (red).
