# Supplementary material for: Combined QTL and Selective Sweep Mappings with Coding SNP Annotation and cis-eQTL Analysis Revealed PARK2 and JAG2 as New Candidate Genes for Adiposity Regulation
Source: G3 (Bethesda). 2015 Feb 3;5(4):517–29. doi: 10.1534/g3.115.016865 (PMC4390568; doi:10.1534/g3.115.016865)
Supplement: Supporting Information [file supp_g3.115.016865_TableS1.pdf]

Table S1 Description of primers used for SNP validations by Sanger re-sequencing.

ID	Sequence	Targeted strand	Start Position[1]	Length	Tm (°C)
**JAG2-Fwd1**	ACCAGCAGATTCCAGTGCCAC	+	1	20	64
**JAG2-Fwd2**	TCAGTATCTCATGTCAAGTGAC	+	411	22	58
**JAG2-Fwd3**	AGTACAAGAAATGCATGGTTCC	+	844	22	59
**JAG2-Fwd4**	ACAGTTCTTCTGGTTGTTAAGG	+	1271	22	59
**JAG2-Fwd5**	TGCTTCTCAGTCTTTCTTTCAC	+	1735	22	59
**JAG2-Rev1**	GCCCCACTAAAACATGAGGG	-	1	20	59
**JAG2-Rev2**	AAGCTAACTCCTTAACAACCAG	-	517	22	58
**JAG2-Rev3**	TGGCTTACTGTAAAGCATCAACTG	-	930	24	62
**JAG2-Rev4**	AATGTCTGGTCATAAGTAGCAG	-	1333	22	58
**JAG2-Rev5**	CAATCAAGCAATTGATCGTG	-	1759	20	56

[1] Relatively to the 3' end of the targeted PCR amplicon.
