# Supplementary material for: Combined QTL and Selective Sweep Mappings with Coding SNP Annotation and cis-eQTL Analysis Revealed PARK2 and JAG2 as New Candidate Genes for Adiposity Regulation
Source: G3 (Bethesda). 2015 Feb 3;5(4):517–29. doi: 10.1534/g3.115.016865 (PMC4390568; doi:10.1534/g3.115.016865)
Supplement: Supporting Information [file supp_g3.115.016865_TableS2.pdf]

Tables S2 Description of primers used for RT-qPCR.

ID	Species	Sequence (5' -> 3')	Length	Tm (°C)
**GG-JAG2-F**	*Gallus gallus*	AAATGCAATCACCAAGCGGC	20	65
**GG-JAG2-R**	*Gallus gallus*	ACCGCACAAAGAATTGGAACC	21	65
**GG-PARK2-F**	*Gallus gallus*	AGCACACCCAACAACTGACA	20	60
**GG-PARK2-R**	*Gallus gallus*	GAGTCTGGACAGCCAGCTAC	20	58
**GG-MLLT4-F**	*Gallus gallus*	ACTGCCACAACTCAGGATGT	20	58
**GG-MLLT4-R**	*Gallus gallus*	TTCAGGGCCATTACTCTGAGC	21	60
**GG-GAPDH-F**	*Gallus gallus*	GCTAAGGCTGTGGGGAAAGT	20	61
**GG-GAPDH-R**	*Gallus gallus*	TCAGCAGCAGCCTTCACTAC	20	60
**MM-PARK2-F**	*Mus musculus*	ATGAATCACAGCCTGCATTGTG	22	62
**MM-PARK2-R**	*Mus musculus*	TACCACCACACACACAGACTTC	22	60
**MM-HPRT-F**	*Mus musculus*	TGGCCATCTGCCTAGTAAAGC	21	62
**MM-HPRT-R**	*Mus musculus*	GGACGCAGCAACTGACATTTC	21	63
