# Supplementary material for: Combined QTL and Selective Sweep Mappings with Coding SNP Annotation and cis-eQTL Analysis Revealed PARK2 and JAG2 as New Candidate Genes for Adiposity Regulation
Source: G3 (Bethesda). 2015 Feb 3;5(4):517–29. doi: 10.1534/g3.115.016865 (PMC4390568; doi:10.1534/g3.115.016865)
Supplement: Supporting Information [file supp_g3.115.016865_TableS3.pdf]

Table S3 Description of primers used for pyro-sequencing-based allelic imbalance analyses.

Chromosome	SNP position	Forward primer	Reverse primer	Sequencing primer
**3**	46581638	[BTN]--GTGCAGCTAGTTACAACAG	CCTTGCCAAAGTGAGTGTT	TTACATTCATTGCATCG
**3**	46581695	[BTN]--GTGCAGCTAGTTACAACAG	CCTTGCCAAAGTGAGTGTT	ATTCTTGCAAAAAACATAAA

[BTN]-- Biotinylated on 5' end.
